# PAF enhances MMP-2 production in rat aortic VSMCs via a β-arrestin2-dependent ERK signaling pathway[S]

**DOI:** 10.1194/jlr.M037176

**Published:** 2013-10

**Authors:** Yun H. Kim, Seung J. Lee, Kyo W. Seo, Jin U. Bae, So Y. Park, Eun K. Kim, Sun S. Bae, Jae H. Kim, Chi D. Kim

**Affiliations:** * Departments of Pharmacology and Pusan National University, Yangsan, Gyeongnam 626-870, Republic of Korea; § Physiology, Pusan National University, Yangsan, Gyeongnam 626-870, Republic of Korea; † School of Medicine, and Medical Research Center for Ischemic Tissue Regeneration, Pusan National University, Yangsan, Gyeongnam 626-870, Republic of Korea

**Keywords:** platelet-activating factor, platelet-activating factor receptor, β-arrestin2, extracellular signal-regulated kinase, matrix metalloproteinase-2

## Abstract

Platelet-activating factor (PAF), 1-O-alkyl-2-acetyl-*sn*-glycero-3-phosphocholine, is a potent phospholipid mediator and has been reported to be localized in atherosclerotic plaque. However, its role in the progression of atherosclerosis remains unclear. In the present study, we investigated the role of PAF in the production of matrix metalloproteinase (MMP) in primary vascular smooth muscle cells (VSMCs). When rat aortic primary VSMCs were stimulated with PAF (1 nmol/l), the expressions of MMP-2 mRNA and protein, but not of MMP-9, were significantly increased, and these upregulations were markedly attenuated by inhibiting extracellular signal-regulated kinases (ERKs) using molecular and pharmacological inhibitors, but not by using inhibitors of p38 mitogen-activated protein kinase or c-Jun N-terminal kinase. Likewise, ERK phosphorylation was markedly enhanced in PAF-stimulated VSMCs, and this was attenuated by WEB2086, but not by EGF receptor inhibitor, demonstrating the specificity of PAF receptor (PAFR) in PAF-induced ERK phosphorylation. In immunofluorescence studies, β-arrestin2 in PAF-stimulated VSMCs colocalized with PAFR and phosphorylated ERK (P-ERK). Coimmunoprecipitation results suggest that β-arrestin2-bound PAFRs existed as a complex with P-ERK. In addition, PAF-induced ERK phosphorylation and MMP-2 production were significantly attenuated by β-arrestin2 depletion. Taken together, the study shows that PAF enhances MMP-2 production in VSMCs via a β-arrestin2-dependent ERK signaling pathway.

Platelet-activating factor (PAF) has been reported to be present in human atherosclerotic plaque, and to induce the production of reactive oxygen species and elastase in macrophages that degrade the extracellular matrix of intima (1). Furthermore, PAF is known to act as an initial trigger in atherosclerosis, and to play a critical role during disease progression (2, 3). The biological actions of PAF are regulated by a specific surface 7-transmembrane-domain receptor (7TMR), which couples to G proteins (4). However, although the PAF receptor (PAFR) is known to be present in smooth muscle cells, the functional significance of intracellular PAFR has not been clarified (5).

Numerous G protein-coupled receptors (GPCRs) activate mitogen-activated protein kinase (MAPK) after specific agonist stimulation (6–8), and after agonist binding, 7TMR phosphorylates MAPKs via the classical G protein-induced synthesis of various second messengers, which include Ca^2+^, or via nonclassical pathways by modulating novel effectors independent of G protein (9). It is also known that stimulation of many GPCRs can activate signaling by receptor tyrosine kinases including epidermal growth factor receptor (EGFR) via a process termed transactivation, and that this leads to the inductions of different signal transduction pathways (10).

Extracellular signal-regulated kinase (ERK) activated by 7TMR may translocate from the cytosol to the nucleus where it activates transcription factors that modulate transcription programs such as that of cAMP response element binding protein (11). On the other hand, phosphorylated ERK (P-ERK) retained in cytosol activates various cytosolic substrates to modulate cell shape and motility (12). Moreover, it has recently been found that 7TMR can use multifunctional adaptor proteins such as β-arrestins to activate many substrates in cellular pathways (13). Initially, β-arrestin was found to bind activated GRK-phosphorylated 7TMRs and thus block or “desensitize” G protein activation induced by agonist stimulation (14). β-Arrestin acts as a bifunctional cellular mediator, that is, it not only terminates G protein signaling, but can also function as a scaffold for the signal transduction of MAPKs including ERK (15, 16).

Matrix metalloproteinases (MMPs), especially MMP-2 and MMP-9, destabilize lesions and erode the fibrous cap of atherosclerotic plaque by digesting extracellular matrix proteins (17). In addition, MMP-2 can function as a mediator of vascular structural changes associated with physiologic and pathologic processes (18). In vivo studies have shown that MMP-2 expression in vascular smooth muscle cell (VSMCs) is associated with various pathologic conditions, especially with atherosclerotic plaque, which exhibit significantly enhanced MMP-2 expression and activation in vulnerable regions (19, 20), thus suggesting MMP-2 plays a critical role in the fate of atherosclerotic plaque. To determine the importance of PAF in the progression of atherosclerosis, we investigated the roles of PAF and PAFR on the production of MMPs in rat aortic primary VSMCs. In addition, we sought to identify the signal pathways involved in PAF-mediated MMP production in VSMCs.

## MATERIALS AND METHODS

### Chemicals and antibodies

PAF, norepinephrine (NE), Bay11-7082, and gelatin were purchased from Sigma (St. Louis, MO). The various signal pathway inhibitors were purchased from Calbiochem (La Jolla, CA). MMP-2 antibody, MAPK antibodies, and phosphospecific antibodies against MAPKs were from Cell Signaling Technology (Beverly, MA). β-Arrestin2 and PAFR antibodies were purchased from Santa Cruz Biotechnology Inc. (Santa Cruz, CA). Calponin and α-smooth muscle actin antibodies were purchased from Sigma. Horseradish peroxidase (HRP)-conjugated IgG (Santa Cruz Biotechnology) was used as the secondary antibody.

### VSMC culture

All animal procedures were performed in accordance with our institutional guidelines for animal research and approved by our institutional animal care and use committee. VSMCs were isolated by enzymatic dissociation from the aortas of 7- to 8-week-old male Sprague-Dawley rats (Charles River Breeding Laboratories, Kingston, NY). Briefly, aortas were dissected, cut into ∼1 mm^2^ segments, and then placed as explants in cell culture dishes containing DMEM (Gibco BRL, Grand Island, NY) with 10% fetal bovine serum (FBS). VSMC purity was determined by staining with smooth-muscle-specific-actin monoclonal antibodies (Sigma). Cells were maintained in DMEM containing 10% FBS at 37°C in a 5% CO_2_ incubator. Human aortic VSMCs, obtained from American Type Culture Collection, were maintained using a growth medium kit (American Type Culture Collection) at 37°C in a 5% CO_2_ incubator.

### Western blot analysis

Protein levels of MMP-2, β-arrestin2, and phosphorylated MAPKs were determined by Western blotting. Briefly, cell lysates of VSMCs were separated on 8% sodium dodecyl sulfate (SDS)-polyacrylamide gels, transferred electrophoretically onto nitrocellulose membranes, which were then blocked with 5% skim milk and incubated with primary antibodies in blocking buffer. Blots were incubated with HRP-conjugated secondary antibody and chemiluminescence intensities were measured using a LAS-3000 SYSTEM (Fuji Photo Film, Japan). Membranes were reblotted with anti-β-actin antibody (MP Biomedicals, Aurora, OH) for control purposes.

### Gelatin zymography

To assess MMP-2 activities, extracellular medium from cultured VSMCs was collected, concentrated 30-fold using a Vivaspin2 Centricon (Sartorius Biolab products, Sartorius AG) and electrophoretically separated in 8% SDS-polyacrylamide gels containing 1 mg/ml gelatin. After washing with wash buffer (2.5% Triton X-100 in 50 mM Tris-HCl, pH 7.4), gels were stained with 0.2% Coomassie Brilliant Blue R-250 (Sigma) for 2 h and then destained in the same solution without dye. Zymographic results, which were obtained using UN-SCAN-IT gel (version 5.1, Silk Scientific, Orem, UT), are expressed as MMP proteolytic activities.

### Measurement of mRNA expression

The expressions of MMP-2 and MMP-9 mRNA in VSMCs were quantified by RT-PCR, using GAPDH mRNA as an internal standard. Total RNA in cultured cells was isolated using Trizol reagent (Invitrogen, San Diego, CA) and reverse transcribed into cDNA using the ImProm-II reverse transcription system (Promega). Amplification of cDNA by PCR was performed using specific primers for MMP-2 (forward, 5′-GTCTTCCCCTTCACTTTTCTG-3′ reverse, 5′-CGGAAGTTCTTGGTGTAGGTG-3′) and MMP-9 (forward, 5′-AAGGATGGTCTACTGGCACAC-3′ reverse, AGAGATTCTCACTGGGGCAGA-3′).

### Measurement of MMP-2 promoter activity

The 5′-flanking promoter region from mouse genomic DNA was amplified by PCR using the upstream primer 5′-AAGGTG*GCTAGC*TCCGTAACGTAGTAG-3′ and the downstream primer 5′-ATCTAA*AGATCT*GGATGCACACAGAGC-3′ the *Nhe*I and *Bgl*II restriction enzyme sites are in italic. Primers were designed using sequences retrieved from GenBank accession numbers NM008610 and BC070430. The amplified 1,584 bp fragment so obtained was cloned into pGL3 basic vector (pGL3-MMP-2), and the identities of the resulting constructs were verified by restriction enzyme digestion and sequence analysis. Transcription factor binding sites within the 5′-flanking promoter region were analyzed using a sequence motif search program from GenomeNet.

Cis-reporter plasmid DNA was prepared using a QIAprep Spin Miniprep kit (Qiagen, Valencia, CA). After cells had been transiently transfected with MMP-2 luciferase reporter plasmids using Lipofectamine 2000 (Invitrogen), luciferase activities in cell lysates were determined using a dual luciferase reporter assay system and a Glomax 20/20 luminometer (Promega).

### Preparation of small interfering RNA and in vitro transfection

Small interfering RNAs (siRNAs) for ERK and universal negative control (NC) siRNA were purchased from Invitrogen (Invitrogen). β-Arrestin2 siRNA was purchased from Santa Cruz Biotechnology. For siRNA transfection, cells were seeded in 6-well plates and grown for 24 h until they reached 40–50% confluence. Cells were then transfected with ERK siRNA, β-arrestin2 siRNA, or NC siRNA using Lipofectamine 2000 (Invitrogen), according to the manufacturer's instructions.

### Immunofluorescence analysis

VSMCs plated on glass coverslips were fixed with 4% paraformaldehyde, and nonspecific binding sites were blocked with 1% BSA. The fixed cells were then incubated with specific antibodies, washed in PBS, and incubated with Alexa488-conjugated IgG and Alexa594-conjugated IgG (Invitrogen). DNA was stained with 0.1 μg/ml diamidino-2-phenylindole in PBS for 3 min at room temperature. Cells were then mounted in carbonate buffered glycerol, and evaluated under a scanning confocal microscope (LSM 510, Carl Zeiss Inc.).

### Coimmunoprecipitation and Western blot analysis

For coimmunoprecipitation assays, cells were lysed with essentially the same buffer, described by Kim et al. (21). Briefly, cleared cell extracts were mixed with 2 μg of PAFR-agarose and incubated for 2 h. Immunoprecipitates were washed with lysis buffer three times and mixed with sample buffer. Samples were then analyzed by Western blotting as described above.

### Statistical analysis

Results are expressed as mean ± SEM. Concentration-dependent responses were analyzed by one-way ANOVA followed by Bonferroni's correction for comparisons of multiple groups. *P* values of <0.05 were considered significant.

## RESULTS

### PAF-induced MMP-2 production in different phenotypes of VSMCs

**Fig. 1.**
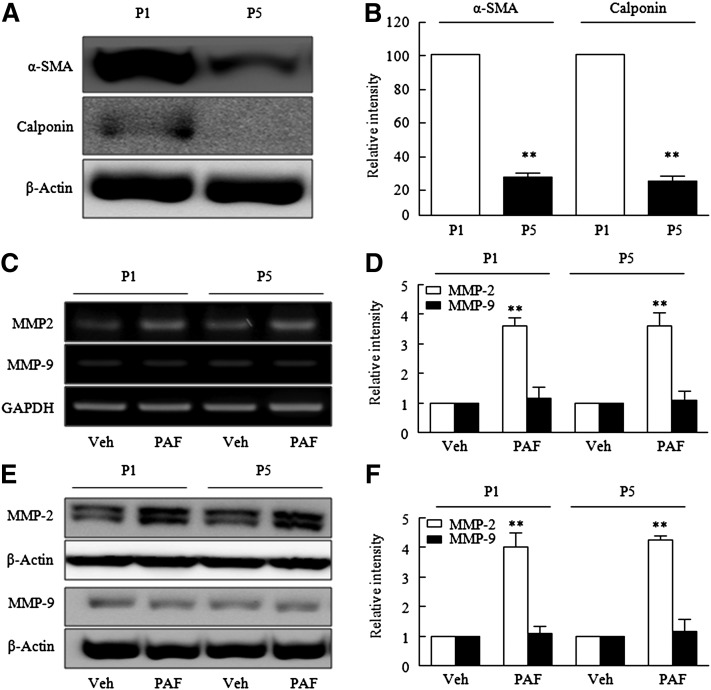
PAF-induced MMP-2 production in different phenotypes of VSMCs. A: Proteins were extracted from early- (P1) and late-passage (P5) VSMCs, and subjected to Western blotting with the differentiation markers α-SMA and calponin. C, E: P1 and P5 VSMCs were stimulated with 1 nmol/l PAF for 12 h. The mRNA and protein expressions of MMP-2 and MMP-9 in PAF-stimulated VSMCs were analyzed by RT-PCR and Western blotting, respectively. B, D, F: Results are presented as the mean ± SEM of four to six independent experiments. **P < 0.01 versus corresponding P1 or vehicle (Veh) values.

Based on the fact that the VSMC phenotype present in atherosclerotic lesions differs from that in regular media, we prepared two types of VSMCs to express different differentiation markers. As shown in **Fig. 1A, B**, α-SMA and calponin were highly expressed in early passage [passage 1 (P1)] VSMCs, but not in late passage [passage 5 (P5)] cells, indicating that VSMC phenotype transited from the differentiated to the dedifferentiated phenotype during passage progression. When VSMCs were stimulated with PAF (1 nmol/l), MMP-2 mRNA (at 6 h), and MMP-2 protein (at 12 h), productions in P1 and P5 VSMCs were significantly increased, whereas MMP-9 showed little change (Fig. 1C–F). On the other hand, PAF-induced MMP-2 production in P5 VSMCs was similar to that in P1 VSMCs. Accordingly, we used late-passage cells (P5) which expressed much lower levels of differentiation markers than early-passage cells in the following study.

### Time-course of PAF-induced MMP-2 production in VSMCs

**Fig. 2.**
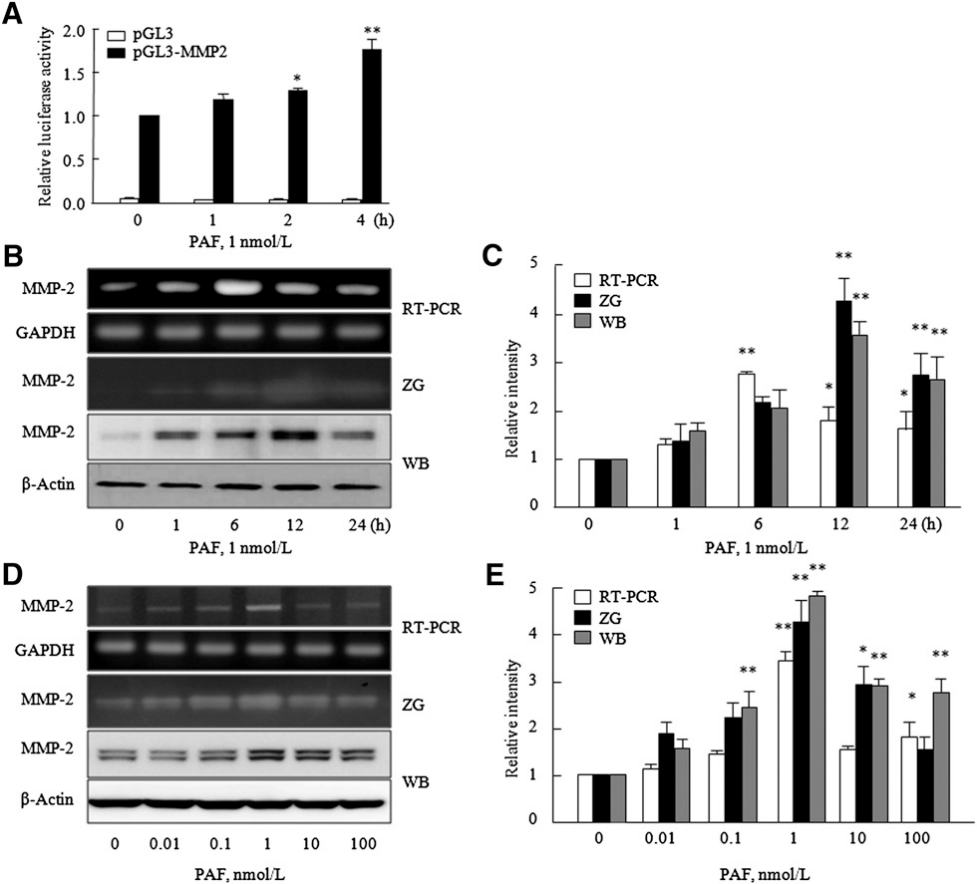
Time-course of PAF-induced MMP-2 production in VSMCs. A: VSMCs were transfected with MMP-2 promoter-luciferase construct (pGL-MMP-2) or empty luciferase vector (pGL3) for 24 h and then stimulated with PAF for the indicated times. MMP-2 promoter activities are expressed as relative luciferase activities, and results are presented as the mean ± SEM from six independent experiments. *P < 0.05, **P < 0.01 versus values at time 0. B: VSMCs were stimulated with PAF for the indicated times (0–24 h). D: VSMCs were stimulated with various concentrations of PAF (0–100 nmol/l). The expressions of MMP-2 mRNA (6 h) were determined by RT-PCR, and MMP-2 activities in extracellular medium and protein levels (12 h) were analyzed by gelatin zymography (ZG) and Western blotting (WB), respectively. C, E: Results are presented as mean ± SEM of five to six independent experiments. *P < 0.05, **P < 0.01 versus corresponding values at time 0 or at zero (0) concentration.

To investigate MMP-2 promoter activity in PAF-stimulated VSMCs, cis-reporter plasmids were transfected into VSMCs and reporter activity was measured. MMP-2 promoter activity in PAF-stimulated VSMCs started to increase at 1 h, and was significantly higher after stimulation for 2 h (**Fig. 2A**). As shown in Fig. 2B, C, MMP-2 mRNA expression and protein production increased time-dependently and peaked after 6 h and 12 h of PAF treatment, respectively. The gelatinolytic activities of MMP-2 were also time-dependently increased by 1 nmol/l of PAF and peaked (3.9 ± 0.37-fold, *P* < 0.01) at 12 h (Fig. 2B, C). In addition, when VSMCs were treated with increasing concentrations of PAF (0–100 nmol/l), MMP-2 mRNA and protein levels increased in a dose-dependent manner up to a PAF concentration of 1 nmol/l. Gelatin zymography showed a concentration-dependent increase in MMP-2 activity which peaked at a PAF concentration of 1 nmol/l (4.3 ± 0.51-fold, *P* < 0.01) (Fig. 2D, E). However, the MMP-9 mRNA expression and protein production were unaffected by PAF (see supplementary Fig. I).

### Involvement of the ERK signaling pathway in PAF-induced MMP-2 production

**Fig. 3.**
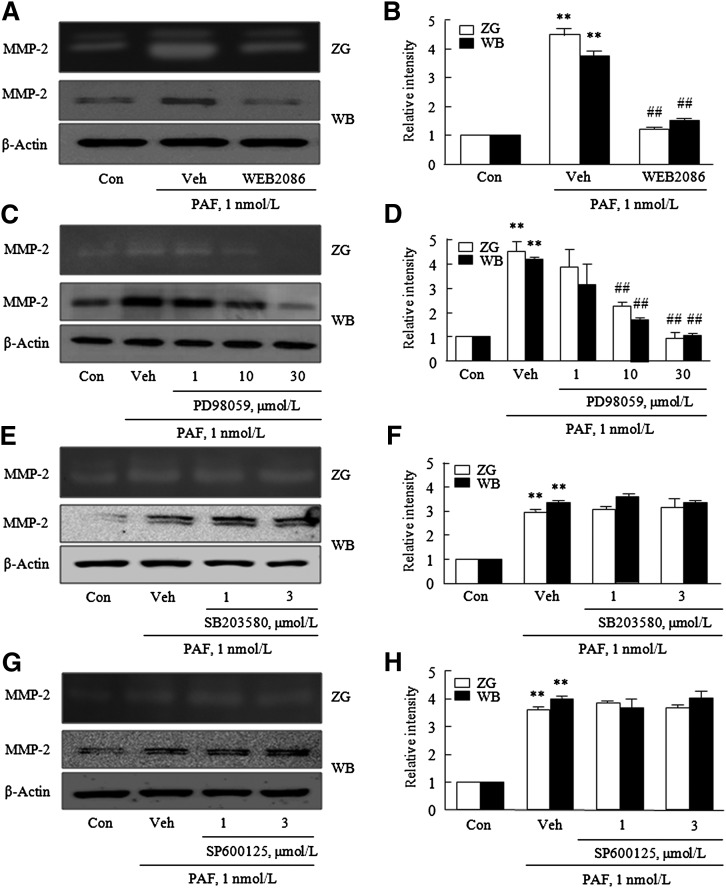
Effects of various signaling inhibitors on MMP-2 activity and production enhancements by PAF. VSMCs were pretreated with WEB2086 (A) for 1 h (2 mmol/l) and with the MAPK inhibitors PD98059 (an ERK inhibitor) (C), SB203580 (a p38 MAPK inhibitor) (E), or SP600125 (a JNK inhibitor) (G) for 30 min, and then stimulated with 1 nmol/l PAF for 12 h. MMP-2 activities in extracellular medium and protein levels were analyzed by gelatin zymography (ZG) and Western blotting (WB), respectively. B, D, F, H: Blots on the left are quantified, and relative intensities are presented as the mean ± SEM of three to five independent experiments. **P < 0.01 versus corresponding controls (Con). ^##^*P* < 0.01 versus vehicle (Veh) controls.

PAF-stimulated MMP2 production and activity were significantly inhibited by 2 mmol/l of WEB2086 (a PAFR antagonist) (**Fig. 3A, B**), indicating that PAFR plays a pivotal role in PAF-induced MMP2 production in VSMCs. To further assess the involvement of MAPK in PAF-induced MMP-2 production in VSMCs, cells were pretreated with inhibitors of MAPKs, that is, PD98059 (an ERK inhibitor), SB203580 (a p38 MAPK inhibitor), or SP900125 [a c-Jun N-terminal kinase (JNK) inhibitor], for 30 min, and then stimulated with PAF (1 nmol/l) for 12 h. As shown in Fig. 3, PAF-induced MMP-2 production was significantly attenuated by PD98059, but not by SB203580 or SP900125. Moreover, PAF-induced MMP-2 production was unaffected by other signaling inhibitors, namely, Bapta (a Ca^2+^ chelating agent), LY294002 (a PI3K inhibitor), or Bay 11-7082 [a nuclear factor κB (NF-κB) inhibitor] (see supplementary Fig. II), which suggested involvement of the ERK signaling pathway in MMP-2 induction by PAF.

**Fig. 4.**
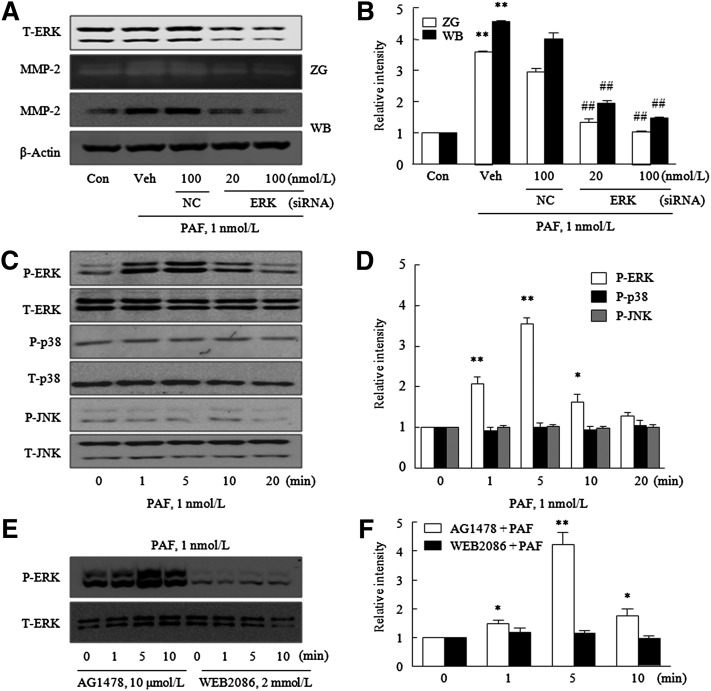
Involvement of the ERK signaling pathway in MMP-2 activity and production enhancements by PAF. A: VSMCs were transfected with the indicated doses of ERK siRNA or NC for 24 h, and total-ERK (T-ERK) levels were determined by Western blotting. MMP-2 activities in extracellular medium and protein levels were determined by gelatin zymography (ZG) and Western blotting (WB), respectively. C: VSMCs were stimulated with PAF for the indicated times, and levels of phosphorylated and total MAPKs were determined by Western blotting. E: VSMCs were pretreated with the indicated doses of AG1478 or WEB2086 for 30 min and 1 h, respectively, and then stimulated with PAF for 12 h. Levels of P-ERK and T-ERK were determined by Western blotting. B, D, F: Blots on the left are quantified, and relative intensities are expressed as the mean ± SEM of three to four independent experiments. *P < 0.05, **P < 0.01 versus corresponding values at time 0 or control (Con). ^##^*P* < 0.01 versus vehicle (Veh) controls.

Accordingly, to confirm that PAF-induced MMP-2 production is mediated via ERK signaling pathways, we transfected VSMCs with ERK siRNA targeting ERK1/2. It was found that VSMCs transfected with siRNA ERK (20–100 nmol/l) showed substantial reductions (0.45 ± 0.05-fold, n = 4) in total ERK expression (**Fig. 4A**). Furthemore, PAF-induced MMP-2 production and gelatinolytic activity were significantly decreased by ERK depletion (Fig. 4A, B), indicating the involvement of ERK pathways in PAF-induced MMP-2 expression.

### Interaction between PAFR and ERK phosphorylation

We also investigated the time-course of the effect of PAF on the phosphorylations of ERK, p38, and c-Jun N-terminal kinase (JNK). Upon pretreatment with PAF (1 nmol/l), P-ERK levels rapidly increased from 1 min, peaked at 5 min, and thereafter declined. However, P-p38 and P-JNK levels showed little change up to 20 min (Fig. 4C, D). To determine whether EGFR transactivation is involved in PAF-induced ERK phosphorylation, VSMCs were pretreated with either AG1478 (an EGFR inhibitor) or WEB2086, and then stimulated with PAF (1 nmol/l). As shown in Fig. 4E, F, ERK phosphorylation in PAF-treated cells was significantly attenuated by WEB2086, but not by AG1478, thus demonstrating PAFR participates in PAF-induced ERK phosphorylation in VSMCs.

### Role of PAF on the subcellular distributions of PAFR, β-arrestin2, and P-ERK

**Fig. 5.**
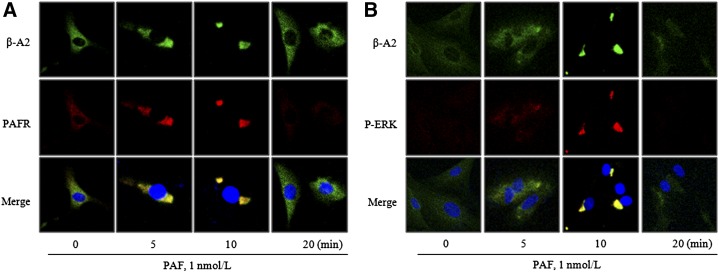
Effects of PAF on the subcellular distributions of PAFR, β-arrestin2, and p-ERK. VSMCs were treated with 1 nmol/l PAF for the indicated times, and then stained with anti β-arrestin2 (β-A2) (top panels, 488 nm), anti- PAFR (A) or anti p-ERK (B) (middle panels, 594 nm). Confocal images of β-arrestin2 were merged with images of PAFR (A) or p-ERK (B) on diamidino-2-phenylindole-stained images (bottom panels). Images are representative of five to six independent experiments.

The immunofluorescence study showed that β-arrestin2 colocalized with PAFR after 5 min of PAF treatment in VSMCs, and that P-ERK colocalized with β-arrestin2 at 10 min. These observations suggest that β-arrestin2 and PAFRs might be complexed with P-ERK after 5 to 10 min of exposure to PAF (**Fig. 5**). In addition, we duplicated these findings in human aortic VSMCs. As shown in supplementary Fig. 3C, D, β-arrestin2 colocalized with PAFR after 5 min of PAF stimulation. Furthermore, P-ERK in VSMCs was activated after 5 min of PAF treatment, and P-ERK colocalized with β-arrestin2 after 10 min. In addition, PAF-induced MMP-2 production was attenuated by PD98059 in human VSMCs (see supplementary Fig. 3A, B).

**Fig. 6.**
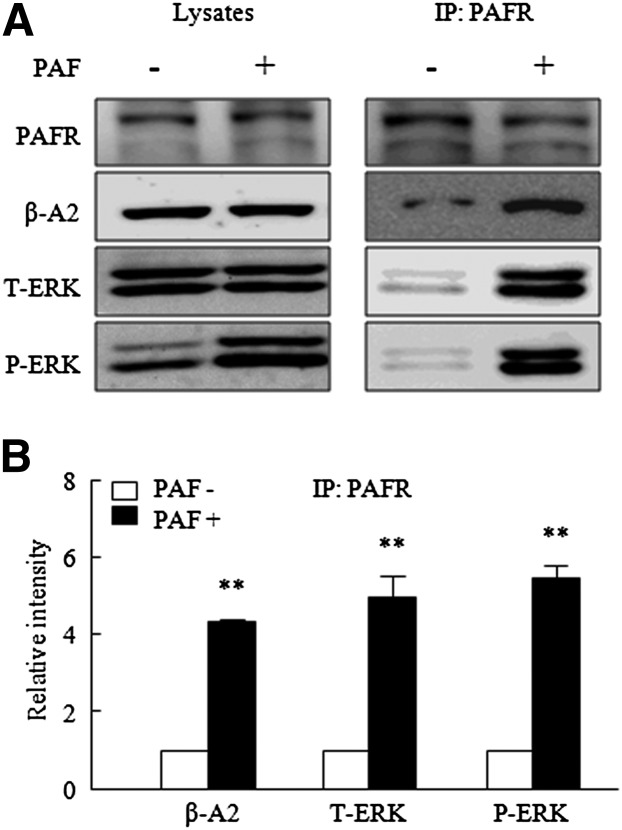
Coimmunoprecipitation of PAFRs, β-arrestin2, and ERK from VSMCs. A: VSMCs were treated with 1 nmol/l PAF for 10 min. Cell lysates were immunoprecipitated (IP) with anti-PAFR antibody and pellets were immunoblotted with anti-ERK, anti-P-ERK, anti-β-arrestin2, or anti-PAFR antibodies. B: Blots were quantified relative to PAFR immunoprecipitated by anti-PAFR antibody, and results are presented as the mean ± SEM of five independent experiments. **P < 0.01 versus corresponding values at no stimulation (−).

We used coimmunoprecipitation to study the effect of PAF on the interaction between PAFR, P-ERK, and β-arrestin2 in VSMCs using PAFR antibody as bait and P-ERK or β-arrestin2 as prey. Interestingly, we detected significantly greater interactions between these proteins in PAF-treated VSMCs than in nontreated controls (**Fig. 6**). These findings suggest that β-arrestin2-bound PAFRs are complexed with P-ERK.

### Role of β-arrestin2 in PAF-induced ERK phosphorylation

**Fig. 7.**
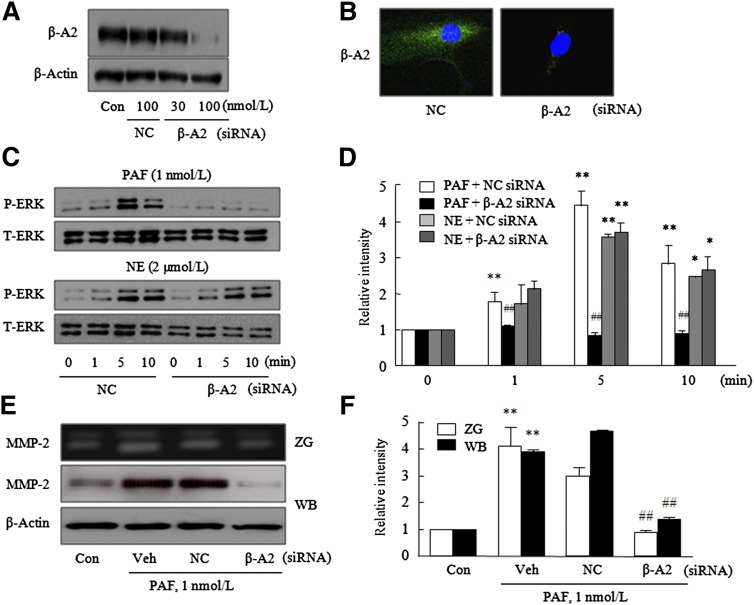
Involvement of β-arrestin2 in ERK phosphorylation and MMP-2 production enhancements by PAF. Representative Western blots (A) and confocal images (B) of β-arrestin2 in VSMCs transfected with the indicated doses of β-arrestin2 (β-A2) siRNA or universal NC for 24 h. C: VSMCs were transfected with β-arrestin2 siRNA (100 nmol/l) or NC for 24 h and then stimulated with PAF (1 nmol/l) or NE (2 μmol/l) for the indicated times. Levels of P-ERK and total-ERK (T-ERK) were determined by Western blotting. D: Blots were quantified, and results are presented as the mean ± SEM of three to five independent experiments. *P < 0.05, **P < 0.01 versus corresponding values at time 0. ^##^*P* < 0.01 versus the NC. E: MMP-2 activities in extracellular medium and protein levels were determined by gelatin zymography (ZG) and Western blotting (WB), respectively. F: Quantitative results are presented as the mean ± SEM of four independent experiments. **P < 0.01 versus corresponding controls (Con), ^##^*P* < 0.01 versus vehicle (Veh) controls.

To investigate the effect of β-arrestin2 on PAF-induced ERK phosphorylation, VSMCs transfected with β-arrestin2 siRNA were stimulated with PAF or NE (an agonist of ERK activation due to G protein activation), and ERK phosphorylations were compared. As shown in Fig. 6C, D, PAF did not increase ERK phosphorylation in VSMCs transfected with β-arrestin2 siRNA, whereas P-ERK levels in β-arrestin2-depleted cells were significantly increased by NE (2 μmol/l) (Fig. 7C, D). In addition, PAF failed to increase MMP-2 activity or production in β-arrestin2-depleted cells (Fig. 7E, F).

## DISCUSSION

The present study identifies the signal pathways by which PAF enhances MMP-2 production in rat aortic primary VSMCs. Increased MMP-2 production in PAF-stimulated VSMCs was attenuated by inhibiting the ERK pathway and by depleting β-arrestin2. In addition, PAF enhanced ERK phosphorylation, and this was significantly inhibited by β-arrestin2 depletion in VSMCs. These results support the hypothesis that PAF enhances MMP-2 production in VSMCs via the β-arrestin2-dependent activation of ERK signaling pathways.

The primary function of VSMCs is contraction. Furthermore, VSMCs have extremely low synthetic activity and a low rate of proliferation (22). However, the extensive plasticity of VSMCs allows them to carry out a number of functions that repair blood vessels, make cells susceptible to a variety of stimuli, and induce phenotypic changes that contribute to the etiologies of cardiovascular diseases (22). Because VSMC phenotypes in atherosclerotic lesions differ from those in regular media of vasculature, we prepared two types of VSMCs that expressed differentiation markers, such as α-SMA and calponin, at different levels. In line with a previous report, which showed significantly decreased α-SMA and calponin levels in late-passage cells (23), the expression of α-SMA and calponin in late-passage (P5) VSMCs was markedly lower than early passage cells, indicating that the VSMC phenotype transited from differentiated to dedifferentiated type during passage progression. However, PAF-induced MMP-2 production in P5 VSMCs was similar to that in P1 VSMCs, therefore, we used late-passage (P5) cells in the following study.

MMP-2 is constitutively expressed in VSMCs in normal arteries, and expressionally upregulated in atherosclerotic arteries (18). In previous in vivo studies, MMP-2 production by VSMCs was found to be linked to a number of pathological conditions, including vulnerable regions in atherosclerotic plaque (24, 25). Reportedly, PAF and its metabolites are key factors during atherosclerotic development (2), and are synthesized by major proinflammatory cells and endothelial cells localized in human atherosclerotic plaques (1). However, because the role of PAF in the progression of atherosclerosis is unclear, we tried to elucidate the role played by PAF in the production of MMPs in VSMCs. It was found that, extremely low PAF concentration (at 1 nmol/l) significantly enhanced MMP-2 production in VSMCs.

Among MAPKs, the application of low PAF at a concentration of 1 nmol/l caused a significant increase in ERK phosphorylation, but in those of p38 MAPK or JNK. These findings are consistent with a report issued by Zhou, Ibe, and Raj (10), in which PAF at 1 nmol/l did not cause the phosphorylation of p38 in fetal ovine venous VSMCs. Previous studies have reported the importance of ERK pathways in MMP-2 production in IL-1β-stimulated VSMCs (26), and in 4-hydroxynonenal-stimulated VSMCs (27). Likewise, we found that PAF-induced MMP-2 production was significantly attenuated by ERK inhibition using molecular and pharmacological inhibitors, but not inhibitions of p38 MAPK, JNK, calcium, and PI3K pathways, which suggests the ERK signaling pathway is important for PAF-induced MMP-2 production in VSMCs. Previously, in human vascular endothelial cells and corneal epithelial cells, ERK was found to play a role in the regulation of PAF-induced MMP-9 expression and activity via a mechanism requiring NF-κB activation (28, 29). However, our results showed that a specific NF-κB inhibitor did not reduce MMP-2 production induced by PAF in VSMCs, which suggests that increased MMP-2 production by PAF might be independent on the NF-κB pathway. Further experiments are required to identify the transcription factors involved in ERK-dependent MMP-2 production in PAF-stimulated VSMCs.

The signaling mechanisms underlying ERK1/2 activation are complex and may originate from the activations of classical G protein-regulated effectors, from cross-talk between 7TMRs and receptor tyrosine kinases (including the EGF receptor) (10) or from β-arrestin scaffolding directly on 7TMR. Gesty-Palmer et al. (30) reported that some differences in ERK activation are receptor and cell type dependent. In murine embryonic fibroblasts stably expressing PAR2 receptor, ERK1/2 phosphorylation was found to be mediated predominantly by a β-arrestin-dependent mechanism, but in HEK293 cells expressing AT1A angiotensin, β-arrestin-dependent and G protein-dependent mechanisms were found to contribute almost equally to the activation of ERK1/2. In the present study, PAF-induced ERK phosphorylation was significantly attenuated by a PAFR antagonist, but not by an EGF receptor inhibitor, thus indicating the importance of PAFR for PAF-induced ERK phosphorylation in VSMCs.

The intracellular loops and C terminus of GPCRs may interact with arrestins. Furthermore, nonvisual arrestins are required for PAFR internalization after specific agonist stimulation (31). In several G_q_-coupled GPCR systems, P-ERK is translocated to the nucleus via the G protein-dependent pathway, whereas β-arrestin-activated ERKs do not translocate to the nucleus because β-arrestin functions as a scaffold for MEK1 and ERKs (13, 32). In addition, a reduction in the receptor-β-arrestin interaction allows the recycling of the internalized receptor, which allows activated ERKs to translocate to the nucleus (32, 33).

In the present study, we first found that in VSMCs treated with PAF, β-arrestin2-bound PAFRs colocalized with P-ERK. Furthermore, as was expected, these complexes did not exhibit translocation from cytosol to the nucleus. In addition, in β-arrestin-depleted cells, the activation of ERK by NE was evident, but ERK phosphorylation induced in PAF-stimulated VSMCs was markedly attenuated. These results indicate that β-arrestin2 is essential for PAF-induced ERK phosphorylation in VSMCs. These results are well supported by a previous report issued by Tohgo et al. (33), in which P-ERK mediated by β-arrestin was found to be retained in the cytosol.

Taken together, we conclude that PAF-enhanced MMP-2 production in rat aortic primary VSMCs occurs via the activation of a β-arrestin-dependent ERK signaling pathway. We believe these findings provide a basis for the development of an efficient therapeutic strategy to suppress atherosclerotic plaque instability.

## Supplementary Material

Supplemental Data
