# Supplementary material for: PAF enhances MMP-2 production in rat aortic VSMCs via a β-arrestin2-dependent ERK signaling pathway
Source: J Lipid Res. 2013 Oct;54(10):2678–86. doi: 10.1194/jlr.M037176 (PMC3770081; doi:10.1194/jlr.M037176)
Supplement: Supplemental Data [file supp_54_10_2678__index.html]

Platelet activating factor enhances MMP-2 production in rat aortic primary vascular smooth muscle cells via a β-arrestin2-dependent ERK signaling pathway — PAF enhances MMP-2 production in rat aortic VSMCs via a β-arrestin2-dependent ERK signaling pathway — Supplemental Data 

# PAF enhances MMP-2 production in rat aortic VSMCs via a β-arrestin2-dependent ERK signaling pathway

## Supplemental Data

**Files in this Data Supplement:**

- Supplemental Data
