# Supplementary material for: PAF enhances MMP-2 production in rat aortic VSMCs via a β-arrestin2-dependent ERK signaling pathway
Source: J Lipid Res. 2013 Oct;54(10):2678–86. doi: 10.1194/jlr.M037176 (PMC3770081; doi:10.1194/jlr.M037176)
Supplement: Supplemental Data [file supp_M037176_jlr.M037176-1.pdf]

SUPPLEMENTARY DATA

Platelet activating factor enhances MMP-2 production in rat aortic primary vascular smooth muscle cells via a β-arrestin2-dependent ERK signaling pathway

Yun H. Kim, Seung J. Lee, Kyo W. Seo, Jin U. Bae, So Y. Park, Eun K. Kim,

Sun S. Bae, Jae H. Kim, Chi D. Kim*

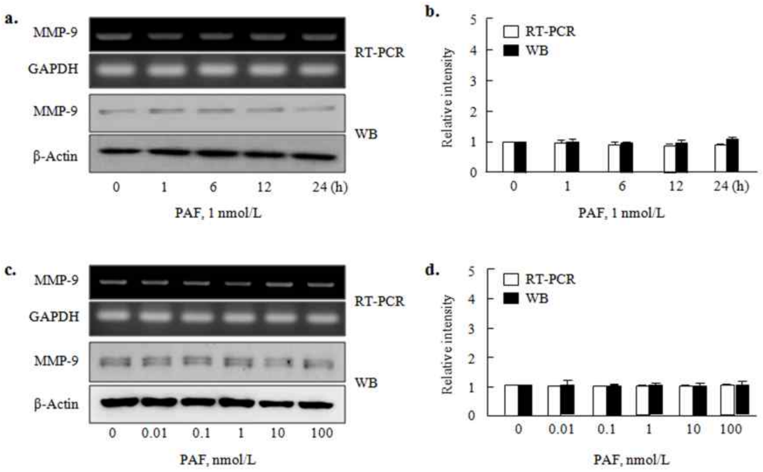

Suppl. Figure 1. MMP-9 production in PAF-treated VSMCs. (a) VSMCs were stimulated with 1 nmol/L PAF for the indicated times (0 – 24 h). (c) VSMCs were stimulated with various concentrations of PAF (0 - 100 nmol/L) for 6 h (for RT-PCR) or 12 h (for Western Blotting). The expressions of MMP-9 mRNA and protein in PAF-stimulated VSMCs were analyzed by RT-PCR and Western Blotting, respectively. (b, d) Quantitative data are presented as the means ± SEMs of 3-5 independent experiments.

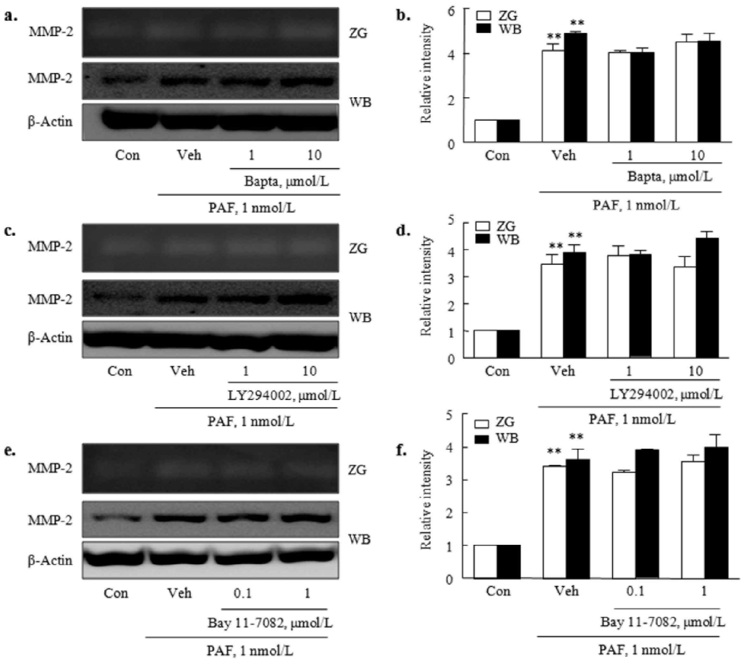

Suppl. Figure 2. Effects of various signaling inhibitors on MMP-2 activity and production enhancements by PAF. VSMCs were pretreated with the indicated doses of inhibitors; (a) Bapta (a Ca^{2+} chelating agent), (c) LY294002 (a PI3K inhibitor), or (e) Bay 11-7082 (a NF-κB inhibitor) for 30 min, and then stimulated with 1 nmol/L PAF for 12 h. MMP-2 gelatinolytic activities in extracellular medium and protein levels were analyzed by gelatin zymography and Western blotting, respectively. (b, d, f) Blot intensities were quantified, and results are presented as the means ± SEMs of 3-5 independent experiments. **P < 0.01, vs. corresponding controls (Con).

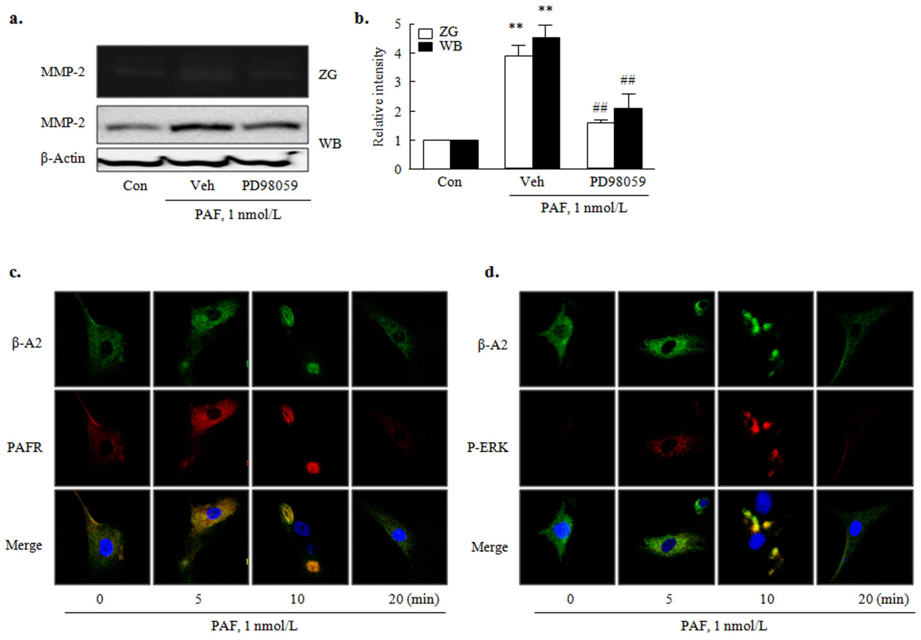

Suppl. Figure 3. Involvement of ERK signaling in PAF-induced MMP-2 production in human VSMCs. (a) VSMCs were pretreated PD98059 (10 μmol/L, an ERK inhibitor) for 30 min and then stimulated with PAF (1 nmol/L) for 12 h. MMP-2 activities in extracellular medium and protein levels were analyzed by gelatin zymography and Western blotting, respectively. (b) Quantitative relative intensities are presented as the means ± SEMs of 3-4 independent experiments. $^{**}P < 0.01$ vs. corresponding controls (Con), $^{\#\#}P < 0.01$ vs. vehicle controls (Veh). (c, d) VSMCs were treated with 1 nmol/L PAF for the indicated times, and then stained with anti β-arrestin2 (β-A2) (*First panels*, 488 nm), anti PAF receptor (PAFR) (a), or anti p-ERK (b) (*Second panels*, 594 nm). Confocal images of β-arrestin2 were merged with images of PAFR (a) or p-ERK (b) on DAPI-stained images (*Bottom panels*). Images are representative of 4-8 independent experiments.
